# Mr. Geoghegan, on Bubo

**Published:** 1805-05-01

**Authors:** Edward Geoghegan

**Affiliations:** Dublin


### *To the Editors of the Medical and Physical Journal.*

Gentlemen,

THAT candour and impartiality which characterize your Journal, induce me to trouble you with a few Observations, in defence of my opinions on the Nature and and Treatment of Bubo, rendered necessary by some remarks in one of your Numbers and in the British Critic.

Practitioners experienced in the treatment of the venereal disease, agree that ulcerated bubo very frequently resists the treatment to which venereal ulcers in other parts as frequently yield; that the sores change their aspect from mildness to malignity, and continue spreading to a dangerous extent, under the use of mercury; that its disuse, in such cases, is attended with beneficial effects, and cures take place without it; whilst in true venereal ulcers of other parts, the reverse invariably occurs; they change from malignity to mildness, and cures are effected by it only.

Mercury, the acknowledged antidote, cures the ulcer every where but in the groin; there, in many instances, it not only does not cure, but aggravates the symptoms; this is not my experience alone. (See the pamphlet.)

Is the Medical Observer, with such facts before his eyes, heedlessly to pass them by, and reject the lessons of experience, insensible to the advantages which the science of medicine derives from a vigilant attention to diseased changes, and the great importance of detecting and marking contra-indications.

The sum of my opinions is, that as mercury is found to aggravate bubo in bad habits, and many recover from the most distressing symptoms without it; there is good reason for abstaining entirely from its use in such habits. Also, it is often doubtful whether or not bubo had a venereal origin, ulcers of the penis, phymosis, and bubo having often occurred, previous to the appearance of the venereal disease, *as noticed in the original work*, is an additional argument in favour of delaying the use of mercury.

I do not consider bubo non-syphilitic in general, nor a critical abscess, but contend that the many cases which occur of its resisting mercury, &c. furnish ground for considering it alone often an equivocal symptom; and as mercury renders it still more equivocal, its exhibition should be delayed until there was more proof that it was necessary. When bubos spread to great extent, and the health is impaired under the influence of mercury in irritable subjects, it is extremely difficult to decide what course is best to be pursued; it would be much less so, if no mercury had been used, and positive evidence existed of the case being venereal.

In my observations I suggested a rationale, which, if well founded, explains the phenomenon under discussion, and gives validity to the opinions I have advanced.

The

The question naturally arises, is it possible that the virus, granting the bubo to have a venereal origin, might proceed no further than the groin? When we consider the structure of glands, it is easy to conceive that an inflamed state would at least suspend for a time the absorption; and that should that state terminate quickly in sloughing, the lymphatics would be destroyed, and in that way the virus be cut of in limine; an event which is admitted as taking place in the phagadenic chancre. Although bubo and constitutional appearances often occur together, still there is abundant evidence that it is frequently local, and to encourage the practice of delaying the use of mercury in such subjects as are known to be materially injured by it. With respect to establishing a general rule, not to give mercury in any case of bubo unaccompanied by other symptoms, I will not insist; but conceive that the peculiar circumstances attending the disease, justify a deviation from the practice ordinarily pursued. I am, &c.

*Dublin, March* 23, 1805. EDWARD GEOGHEGAN

